# Sea Clutter Suppression and Target Detection Algorithm of Marine Radar Image Sequence Based on Spatio-Temporal Domain Joint Filtering

**DOI:** 10.3390/e24020250

**Published:** 2022-02-08

**Authors:** Baotian Wen, Yanbo Wei, Zhizhong Lu

**Affiliations:** 1 College of Intelligent Systems Science and Engineering, Harbin Engineering University, No. 145 Nantong Street, Harbin 150001, China; 20130415@hrbeu.edu.cn; 2 College of Physical and Electronic Information, Luoyang Normal University, No. 6 Jiqing Road, Luoyang 471934, China; weiyanbo@hrbeu.edu.cn

**Keywords:** radar image sequence, sea clutter suppression, target detection, spatio-temporal domain joint filtering, marine radar

## Abstract

In marine radar target detection, sea clutter will cause a large number of missed alarms and false alarms, which will affect the accuracy of target detection. In order to suppress sea clutter effectively, a sea clutter suppression and target detection algorithm of marine radar image sequence based on spatio-temporal domain joint filtering is proposed in this paper. The proposed method is to add a sea clutter suppression link before detecting the target. Firstly, the marine radar image sequence is transformed into three-dimensional frequency wavenumber domain by three-dimensional fast Fourier transform (3D-FFT), and then the three-dimensional image spectrum is obtained. According to the fact that the sea clutter spectrum obtained from the image spectrum satisfies the dispersion relation of linear wave theory in the three-dimensional frequency wavenumber domain, a sea clutter model is established. Then, through the established sea clutter model, a spatio-temporal domain joint sea clutter suppressor is designed to filter the image spectrum. After that, the filtered image spectrum is transformed by three-dimensional inverse fast Fourier transform (3D-IFFT) to obtain the image sequence in which sea clutter is suppressed. Finally, target detection is carried out for sea clutter suppressed image sequence. The method is validated by using the real data of X-band marine radar. Compared with the classical Empirical mode decomposition (EMD) method, the improvement of signal-to-noise ratio (SNR) is more obvious, and SNR can be increased by 15.3 db at most. In addition, compared with target detection on original images directly, the proposed method has excellent detection rate and can increase detection rates by at least 8%.

## 1. Introduction

It is always a challenge for marine radar to detect weak target effectively under the background of complex sea clutter. At the moment, the methods widely used in X-band marine radar target detection are divided into two categories [1]: constant false alarm rate (CFAR) for target detection and track before detect(TBD) for target tracking.

Based on the background clutter statistical model, the CFAR detector adaptively determines the threshold through the known background clutter distribution and the preset false alarm rate [2]. For different sea clutter background distributions, the first proposed CFAR is the cell-averaging CFAR (CA-CFAR). CA-CFAR estimates the gray level of sea clutter by calculating the mean value of the reference unit at both ends of the range unit [3], whereas its detection performance will significantly reduce in multi-target and inhomogeneous environments. Hence, Rohling et al. proposed an order statistics CFAR (OS-CFAR) [4]. It estimates the gray level of sea clutter by orderly arranging the gray values in the reference unit. Although OS-CFAR has achieved good detection results in multi-target and inhomogeneous environments, its detection performance will decline in uniform environments. In order to solve the above problem, Gandhi et al. proposed a trimmed mean CFAR (TM-CFAR) [5]. TM-CFAR improves its detection performance in uniform environment by deleting a certain number of minimum values and maximum values in the sorted reference unit gray value sequence. In the meantime, the Bayesian method was introduced to improve the robustness of the CFAR detector [6]. A Bayesian detector reduces the influence of the interfering targets by modeling and removing them. However, such detectors require appropriate prior information and are computationally complex. Recently, a CFAR based on weighted likelihood (WL) estimator for Weibull clutter with known shape parameter is proposed [7]. WL-CFAR estimates the scale parameters of Weibull distribution by maximizing the weighted log likelihood function. This method has perfect effects in both uniform and inhomogeneous environments. Due to the fact that the CFAR detector only depends on the gray value information of marine radar, this method can only have stable detection effect for targets with high SNR.

Compared with the CFAR method, TBD utilizes multiple original marine radar images to accumulate energy and extract useful energy without threshold detection. Among marine radar TBD methods, the most common filtering methods are Kalman filter (KF) and Particle filter (PF). Chen et al. proposed a sampling importance resampling particle filter (SIR-PF) in [8]. Based on a sequence of practical X-band marine radar images, SIR-PF uses two histogram-based target models that include a kernel-weighted histogram model and a background-weighted histogram model to estimate only target positions. After that, Chen et al. proposed a combined PF-KF approach [1]. In the combined PF-KF approach, PF is used to estimate target coordinates, and then KF further determines the target position and speed according to the results derived from PF. In [9], the motion information of the target is estimated from marine radar images by combining SIR-PF with visual tracking strategies based on template matching. As mentioned earlier, the main idea of TBD is to enhance the target gray value through energy accumulation.

The key problem of radar weak target detection is how to improve SNR. There are two methods to improve SNR. In addition to enhancing the gray value of the target, it can also suppress the sea clutter gray value [10]. The precondition of suppressing sea clutter is that the target and sea clutter can be distinguished in a certain dimension. Therefore, the characteristic differences between target and sea clutter in different dimensions such as the spatio-temporal domain, time-frequency domain and range Doppler domain should be analyzed [11].

The electromagnetic wave emitted by radar radiates on the sea surface to form backscattering echo is defined as sea clutter. Due to the fact that the variation of amplitude of sea clutter is a stochastic process, that is, it is non-stationary in time and inhomogeneous in space, the characteristics of sea clutter are extremely complex. Moreover, sea clutter will be severely affected by wind speed, current velocity, temperature and humidity of the sea surface. As a result, the characteristics of sea clutter in different environments change significantly, which makes it difficult to establish a sea clutter model and suppress sea clutter.

At present, target detection methods under sea clutter suppression are mainly divided into four categories in [12,13,14]: time-domain cancellation method, subspace decomposition method, neural network clutter suppression method and time-frequency analysis. Time-domain cancellation method mainly includes moving target indication (MTI) [15], motion target detection (MTD) [16,17,18,19,20], adaptive moving target indication (AMTI) [21,22], space-time adaptive processing (STAP) [23,24,25,26] and root loop cancellation method [27,28]. The MTI method uses the difference of Doppler frequency between the target signal and the sea clutter signal to design the filter so that the band stop notch of the filter is aligned with the center frequency of sea clutter as much as possible to realize the suppression of the sea clutter. Based on MTI, MTD method connects a group of adjacent and partially overlapping filters to cover the entire Doppler frequency range so as to realize the detection of moving targets with specific Doppler frequency shifts. AMTI adaptively estimates the center Doppler frequency of sea clutter based on MTI. The realization of STAP depends on the accurate estimation of clutter covariance matrix containing Doppler frequency information. The root loop cancellation method uses the time–Doppler frequency information of the signal to estimate the first-order Bragg peak of sea clutter, thereby reconstructing sea clutter in the time domain and subtracting it from the signal to suppress sea clutter [29,30]. These methods need Doppler information of radar signals, and it is not applicable to radar signals without Doppler information.

Subspace decomposition method mainly includes using eigenvalue decomposition [31,32,33,34] and singular value decomposition (SVD) [35,36,37,38] to suppress sea clutter. Among them, the SVD method is a typical research content and a method based on the characteristics of the first-order Bragg peak of sea clutter in the Doppler domain. The Hankel matrix is constructed by using the signal pulse sequence of a certain range bin, and the singular value decomposition of the Hankel matrix is calculated. After that, the signal is decomposed into clutter subspace, signal subspace and noise subspace. At the next stage, the singular value corresponding to clutter subspace is set to zero to obtain the reduced rank Hankel matrix. At last, the reduced rank Hankel matrix is used to reconstruct the time-domain signal, and the sea clutter suppressed time-domain signal can be obtained. Similarly to the time-domain cancellation method, the subspace decomposition method also needs to use the Doppler information of radar signal to suppress sea clutter.

Neural network clutter suppression method includes convolutional neural network [14,39,40,41], radial basis function neural network [13,42,43], wavelet neural network [44,45], etc. The neural network clutter suppression method is to train and optimize itself by using the chaotic characteristics and predictability of sea clutter and, thus, to establish the prediction model of sea clutter. Afterwards, the trained network model is used to predict the unknown sea clutter time series in one or more steps. Even so, this method manually adjusts network structure and parameters according to prior information for different sea clutter data. Moreover, this method requires radar to accumulate pulse for long periods of time in dwell mode to establish a sea clutter prediction model, which is not suitable for radar with short time accumulation pulse in scanning mode.

The time-frequency analysis method uses the principle that sea clutter and target have different time-frequency characteristics in time-frequency domain to suppress sea clutter. Short time Fourier transform [46,47], fractional Fourier transform [48,49,50,51,52] and sparse Fourier transform [53,54,55] all belong to this method. This method transforms the time series data on a range bin into time-frequency domain by using different time-frequency transform. Later, the location of the frequency aggregation of the target signal is found in the time-frequency domain. Afterwards, the target is extracted and the effect of suppressing sea clutter is achieved. In the same manner, this method only has perfect effects on linear frequency modulation signals and needs the radar to accumulate pulse for a long time in dwell mode. In addition, there is another time-frequency analysis that maps one-dimensional time signal to two-dimensional TF plane by using TF joint representation in [56,57,58,59]. Similarly to the time-domain cancellation method, this method also needs to use the Doppler information of the radar signal.

Among the time-frequency analysis methods, there is also a time-domain analysis method applied to sea clutter suppression. The empirical mode decomposition (EMD) [60,61,62,63] method is a local feature analysis method based on time domain. It can extract transient and steady-state information according to the time-domain signal amplitude sequence. However, the performance of EMD is affected by sampling frequency. When sampling frequency is low, the EMD method has excellent suppression effects on sea clutter around the micro-motion target or floating target. Nonetheless, the suppression performance of sea clutter around fast moving targets will decrease, but it is still effective.

The vast majority of marine radar is a slow rotating noncoherent radar with monopulse system. There is only amplitude and position information but lack of phase information in its signal data. In addition, its working mode is scanning, which is different from the dwell mode. In order to take into account scanning efficiency, the scanning mode usually accumulates few pulses in a short period of time on a range bin; thus, marine radar belongs to short-time accumulation radar. Moreover, since the time interval between two consecutive frames is about 2.5s, the time resolution of marine radar is low. Therefore, except the EMD method, the above target detection methods under sea clutter suppression are not suitable for marine radar.

Due to the characteristics of low production cost, simple operation, all-weather work and long working distance, marine radar has become an essential equipment for all types of ships. Marine radar is mainly used to detect objects that affect navigation safety around ships, such as other ships, navigation marks, floating ice and islands. It is an important instrument for mariners to avoid collisions, navigate, locate, observe and rescue [64]. Among them, the detection of unknown floating weak targets on the sea surface is the most important task. However, due to the inherent properties of marine radar, it is extremely difficult to detect weak targets in severe sea clutter interference, and most target detection algorithms under sea clutter suppression are not applicable. Therefore, it is urgent to research a method that only uses signal amplitude information to suppress sea clutter and detect targets.

Inspired by the wave parameter inversion theory of X-band marine radar [65,66,67], aiming at the problem that the existing target detection methods under sea clutter suppression are not applicable to non-coherent marine radars, this paper proposes a sea clutter suppression and target detection algorithm of marine radar image sequence based on spatio-temporal domain joint filtering. In this method, firstly, sea clutter energy in the image sequence is filtered by using the spatio-temporal domain joint sea clutter suppressor. Then, a detector is used to detect the target in the processed image sequence. This method is a new algorithm that can effectively suppress sea clutter signal by using the amplitude information of radar signal and complete high accuracy target detection.

The remainder of this paper is organized as follows. Section 2 analyzes and establishes a theoretical model of sea clutter in the marine radar image sequence. In Section 3, it introduces the algorithm process proposed in this paper. The performance of the proposed algorithm is demonstrated by using real X-band marine radar data in Section 4. Finally, the conclusions are presented in Section 5.

## 2. Sea Clutter Model of Marine Radar Image Sequence

In hydrodynamics, the linear wave theory linearise representations of gravity wave propagation on uniform fluid layer surface. The theory that describes the dynamics and kinematics of wave is frequently used to estimate the characteristics of waves. LeBlond and Mysak found in the study of hydrodynamic characteristics of wave that the gravity wave of sea wave conformed to the dispersion relationship in the linear wave theory. LeBlond et al. assumed that the wave field and current field on the sea surface are spatially uniform and temporally stable in the selected region, and the first-order gravity wave of the sea wave satisfies the following dispersion relationship [65,66,67]:

(1) $$\omega =\sqrt{g\cdot k\cdot \tanh(k\cdot h)}$$

where ω is the frequency of sea gravity wave, *g* is the gravity acceleration, *h* is the water depth and *k* is the modulu of wave number.

**Figure 1:**
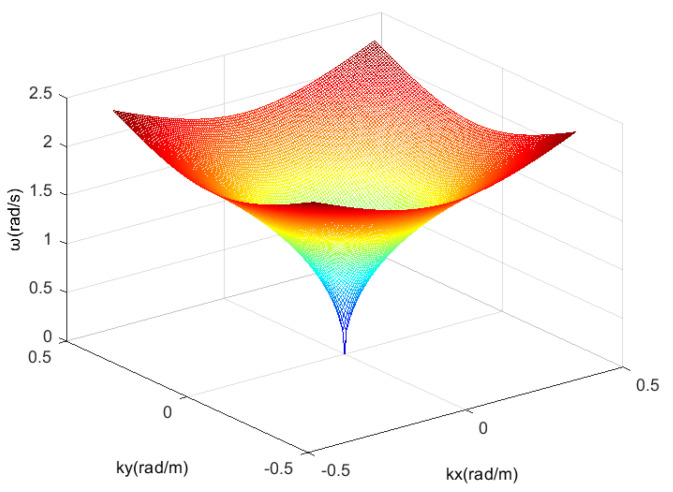
The dispersion relation curved surface; current velocity is 0 m/s.

The premise of the relationship in Equation (1) is that the velocity of the sea surface current is zero. The existence of sea surface current will cause a Doppler frequency shift. Meanwhile, the current $\vec{u}$ will keep the wave number *k* unchanged and offset the original ω, which will influence the image spectrum. Influenced by Doppler frequency shift effect, the image spectrum of the wave for which its propagation direction is consistent with the current direction migrates to a relatively higher frequency position. Correspondingly, the image spectrum of the wave for which its propagation direction deviates from the current direction migrates to a relatively lower frequency position. Therefore, considering the influence of sea surface current in Equation (1), Equation (2) is obtained. Figure 1 shows the dispersion relation curved surface when the current velocity is 0 m/s.

(2) $$\omega =\sqrt{g\cdot k\cdot \tanh(k\cdot h)}+\vec{k}\cdot \vec{u}$$

**Figure 2:**
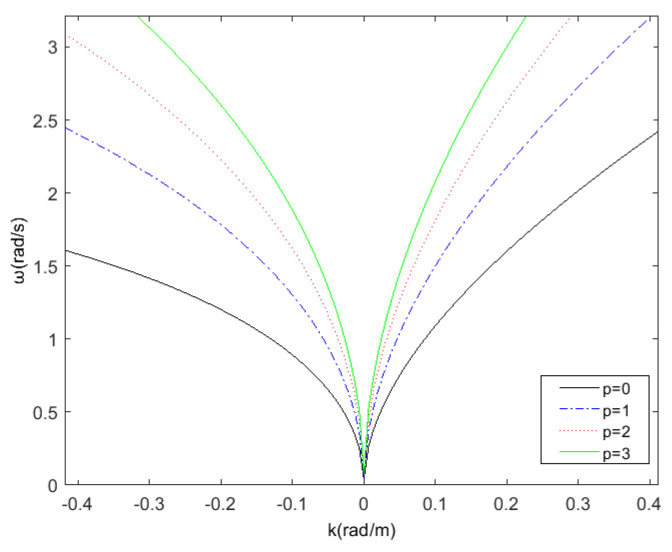
The dispersion relation curves of different order; current velocity is 1 m/s.

Except for the basic dispersion relation satisfied by the first order gravity wave, there are higher-order dispersion relations defined as follows:

(3) $$\omega =\left(p+1\right)\sqrt{g\cdot k\cdot \frac{1}{p+1}\cdot \tanh\left(\frac{k\cdot h}{p+1}\right)}+\vec{k}\cdot \vec{u}$$

where, p=0,1,2..., when *p* is 0, Equation (3) is consistent with the Equation (2). At this moment, the dispersion relation is linear dispersion relation, and the corresponding Equation (2) is the basic dispersion relation equation. When *p* is not 0, the dispersion relation is nonlinear, and Equation (3) is the corresponding p-order dispersion relation equation. Figure 2 shows the dispersion relation curves of different order when the current velocity is 1 m/s; at this moment in time, the set current direction is the same as the positive wave direction. It can be observed from Figure 2 that the higher the order of the dispersion relation, the greater the frequency of the corresponding curve at the same wave number.

**Figure 3:**
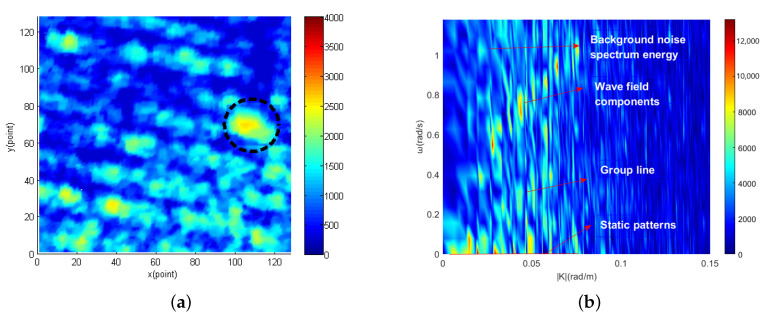
Radar image containing target and 2D cross section of 3D image spectrum $I_{\omega ,k}$ along |K| direction. (**a**) radar image containing target. (**b**) 2D cross section of 3D image spectrum $I_{\omega ,k}$ along |K| direction.

Radar image containing target obtained from the East China Sea are shown in Figure 3a. The part within the black circle in the image is target. The 2D cross section of the three-dimensional image spectral domain along the |K| direction obtained from Figure 3a is shown in Figure 3b. It can be seen from Figure 3b that the image spectrum is composed of different kinds of spectrum. The first kind is the sea clutter spectrum, which mainly concentrates near the basic dispersion relation [65]. The second kind is the background noise spectrum caused by sea surface roughness [68]. The third kind is static and quasi-static signal spectrum. Since the electromagnetic wave will fade when it propagates in the air, the energy will attenuate in the radial distance. Static and quasi-static spectrum are generated in this process and mainly concentrated in the extremely low frequency region of the image spectrum [69]. The fourth kind is the nonlinear wave characteristic spectrum, which is the group line generated by second-order interactions between two different components [70]. The fifth kind is target spectrum scattered in the image spectrum. For moving target, the target spectrum is related to the size and the gray value of the target itself. On the other hand, the spectrum of moving target is not only related to size and gray value but also related to motion speed and motion mode.

## 3. Sea Clutter Suppression and Target Detection Algorithm

### 3.1. Spatio-Temporal Domain Joint Sea Clutter Suppressor

Firstly, three-dimensional Fourier transform is used to transform radar image sequence η(x,y,t) in temporal and spatial domain into frequency wavenumber spectrum $F\left(k_{x},k_{y},\omega \right)$. The image spectrum $I\left(k_{x},k_{y},\omega \right)$ is obtained from the frequency wavenumber spectrum $F\left(k_{x},k_{y},\omega \right)$. The discrete form is as follows:

(4) $$F\left(k_{x},k_{y},\omega \right)=\sum_{0}^{L_{y}}\sum_{0}^{L_{x}}\sum_{0}^{T}\eta \left(x,y,t\right)\exp\left[-2\pi i\left(k_{x}x/L_{x}+k_{y}y/L_{y}+\omega t/T\right)\right]$$

(5) $$I\left(k_{x},k_{y},\omega \right)=\frac{1}{L_{x}L_{y}T}{\left|F\left(k_{x},k_{y},\omega \right)\right|}^{2}$$

where the following is the case.

(6) $$\Delta k_{x}=\frac{2\pi }{L_{x}},\;\Delta k_{y}=\frac{2\pi }{L_{y}},\;\Delta \omega =\frac{2\pi }{T}$$

$L_{x}$ and $L_{y}$ represent the scale of the image sequence in the spatial domain. *T* is the scale of the image sequence in the temporal domain. $I\left(k_{x},k_{y},\omega \right)$ denotes 3D frequency wavenumber image spectrum. $\Delta k_{x}$ and $\Delta k_{y}$ represent the resolution of wave number component. Δω represents the resolution of frequency.

Based on the principle that the energy of sea clutter concentrates around the basic dispersion relationship, a sea clutter suppressor can be designed. In order to reduce the influence of sea current, on the basis of the leading wavelength dispersion relation band pass filter (LDRBF) proposed by R.C. Jia in the inversion of sea wave parameters, a spatio-temporal domain joint sea clutter suppressor was proposed. The designed suppressor is as follows.

Transform the three-dimensional frequency wavenumber image spectrum $I\left(k_{x},k_{y},\omega \right)$ in the rectangular coordinate system into the three-dimensional frequency wavenumber image spectrum $I\left(|K|,\theta_{1},\omega \right)$ in polar coordinates:

(7) $$I\left(|K|,\theta_{1},\omega \right)=I\left(k_{x},k_{y},\omega \right)\cdot \beta$$

where |K| is the module of wave number. $\theta_{1}$ is the image spectrum wave number angle. β is the transformation matrix to the coordinate system.

The image spectrum in 2D frequency wavenumber modulu domain I(|K|,ω) is obtained by integrating $I\left(|K|,\theta_{1},\omega \right)$ with a wave number angle.

(8) $$I\left(|K|,\omega \right)=\int_{0}^{2\pi }I\left(|K|,\theta_{1},\omega \right)d\theta_{1}$$

The maximum spectral value *C* in the 2D image spectrum I(|K|,ω) is calculated, and then the wavenumber modulus of all points for which its spectral value is greater than 0.95∗C is obtained. $\left|K_{m}\right|$ is gained by averaging these wavenumber modulus, which represents the wavelength of the sea clutter with high wave height and a large number in the image sequence. The sea clutter mentioned above is called leading waves. Furthermore, the wavelengths of most of the sea clutter are close to the wavelength of the surrounding leading waves.

In order to deduce the upper and lower bounds of the filter frequency band, it is assumed that sea depth *h* is deep enough to make tanh(|K|h)≈1. Then, the basic dispersion relation equation changed from Equation (2) to the following.

(9) $$\omega =\sqrt{g\cdot |K|}+\left|K\right|\cdot \left|U\right|\cdot \cos\left(\theta_{0}\right)$$

Considering the influence of waveumber resolution and frequency resolution on Equation (9), the upper and lower boundaries are as follows:

(10) $$\omega +\frac{\Delta \omega }{2}-\left|K\right|\cdot \left|U\right|\cdot \cos\left(\theta_{0}\right)\geq \sqrt{g\left(\left|K\right|-\frac{\Delta K}{\sqrt{2}}\right)}$$

(11) $$\omega -\frac{\Delta \omega }{2}-\left|K\right|\cdot \left|U\right|\cdot \cos\left(\theta_{0}\right)\geq \sqrt{g\left(\left|K\right|+\frac{\Delta K}{\sqrt{2}}\right)}$$

where $\Delta K=\sqrt{{\left(\Delta K_{x}\right)}^{2}+{\left(\Delta K_{y}\right)}^{2}}=\sqrt{2}\Delta K_{x}=\sqrt{2}\Delta K_{y}$ is the resolution of wavenumber module. $\theta_{0}$ is the angle between the current direction and the positive direction of wave number.

Bring the leading wave value $\left|K_{m}\right|$ and the maximum relative current velocity $\left|U_{\max}\right|$ into Equations (10) and (11). Take $\cos\left(\theta_{0}\right)=-1$ at the upper boundary and $\cos\left(\theta_{0}\right)=1$ at the lower boundary in order to maximize the band-stop region of the filter. The upper boundary and the lower boundary of the filter can be obtained as Equations (12) and (13):

(12) $$\left|K_{p}\right|=\frac{{\left(\omega +\frac{\Delta \omega }{2}+\left|K_{m}\right|\cdot \left|U_{\max}\right|\right)}^{2}}{g}+\frac{\Delta K}{\sqrt{2}}$$

(13) $$\left|K_{n}\right|=\frac{{\left(\omega -\frac{\Delta \omega }{2}-\left|K_{m}\right|\cdot \left|U_{\max}\right|\right)}^{2}}{g}-\frac{\Delta K}{\sqrt{2}}$$

where $\left|K_{p}\right|$ is the upper boundary of the frequency band-stop of the filter. $\left|K_{n}\right|$ is the lower boundary of the frequency band-stop of the filter.

According to Equations (12) and (13), a mathematical model of the sea clutter suppressor is generated.

(14) $$E\left(k_{x},k_{y},\omega \right)=\left\{\begin{array}{cc} 0 & \omega \in \left[K_{n},K_{p}\right]\\ I\left(k_{x},k_{y},\omega \right) & \;\mathrm{else}\; \end{array}\right.$$

The value of $\left|K_{m}\right|$ in the suppressor is obtained from the wave condition of the image itself without setting parameters. Moreover, $\left|U_{\max}\right|$ is used instead of current estimation. The range of $\left|U_{\max}\right|$ value is determined according to the current data in the experimental sea area and can be taken between 1.2∼3 m/s. In addition, benefit by the large bandwidth, the suppressor can adapt to any sea condition.

**Figure 4:**
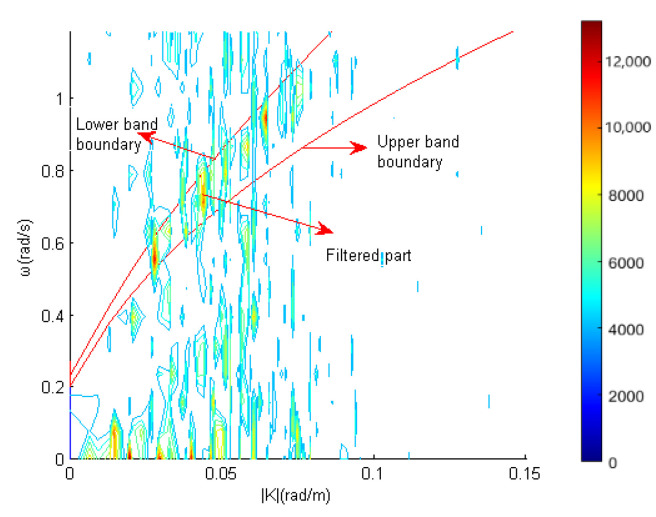
The shape of the suppressor in 2D frequency wavenumber modulo domain.

In order to describe the suppressor more intuitively, Figure 4 shows the shape of the suppressor in 2D frequency wavenumber modulo domain. The image spectrum is derived from marine radar image sequence data of the East China Sea on 20 October 2017.

The three-dimensional spectrum with sea clutter suppressed $\eta_{m}\left(x,y,t\right)$ is transformed by three-dimensional inverse Fourier transform to obtain the sea clutter suppressed image sequence.

(15) $$\eta_{m}\left(x,y,t\right)=\frac{1}{L_{y}}\frac{1}{L_{x}}\frac{1}{T}\sum_{0}^{L_{y}}\sum_{0}^{L_{x}}\sum_{0}^{T}E\left(k_{x},k_{y},\omega \right)\exp\left[2\pi i\left(k_{x}x/L_{x}+k_{y}y/L_{y}+\omega t/T\right)\right]$$

### 3.2. Sea Clutter Suppressed Image Sequence Target Detection

In this paper, weighted likelihood constant false alarm rate(WL-CFAR) [7] is used to detect targets in sea clutter suppressed image sequence. It estimates the scale parameters of Weibull distribution by maximizing the weighted log likelihood function. Then, based on the known Weibull distribution and the preset false alarm rate, it can adaptively determines the threshold.

## 4. Experimental Result

### 4.1. Marine Radar Parameters and Experimental Data

In this paper, real measured X-band marine radar data are used to evaluate the performance of the proposed method. During the experiment, the marine radar collected a large amount of data on the East China Sea in October 2017. X-band marine radar parameters and experimental data radar parameters are shown in Table 1. The rotation time of the radar antenna is 2.5 s, the number of lines generated in a single radar image is 2048, and each line has 600 pixels. In addition, the water depth of the area where the data were collected is about 48 m.

The working mode of experimental marine radar are scanning mode. For a point in a certain azimuth of the image, the residence time of the rotating antenna is given by Equation (16):

(16) $$T=\frac{HBW}{\omega }=\frac{HBW}{RPM\times 360^{\circ }/60\;s}$$

where *HBW* is the horizontal beam width of radar. *RPM* is the revolution per minute of the antenna.

In this experiment, *HBW* provided by the marine radar is 1.3°, *RPM* of the antenna is about 26 and the rotation angular velocity of the antenna is 156°/s. The calculated *T* is 8.3 ms.

According to Equation (17), the number of pulses accumulated at a single point within 8.3ms is calculated, wherein the pulse repetition frequency (*PRF*) is 1300 Hz.

(17) $$N_{P}=T\times PRF$$

The calculated $N_{p}$ is about 11, and because the time interval between two consecutive images is 2.5 s, the number of pulses accumulated by the marine radar in short time is extremely low.

**Figure 5:**
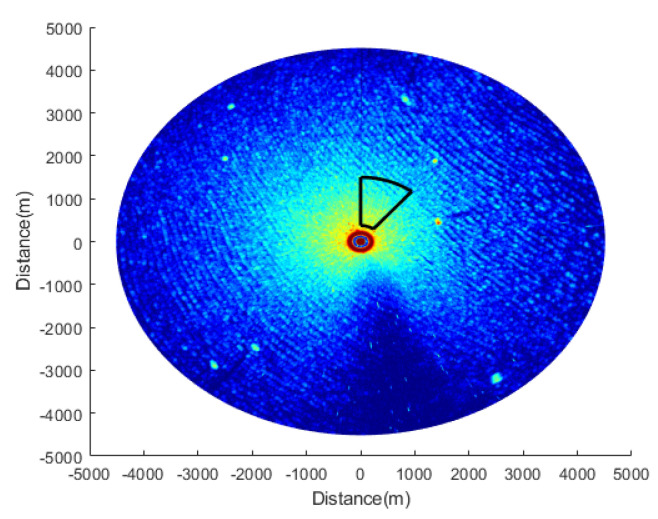
A single X-band marine radar original image.

Figure 5 shows a single radar original image that contains a target signal and sea clutter signal. The maximum value of the gray level of the signal is 8192 in the image. Moreover, the data selected in this paper contain three sea conditions (0.5 m–3 m), which are divided into low sea condition, medium sea condition and high sea condition.

**Figure 6:**
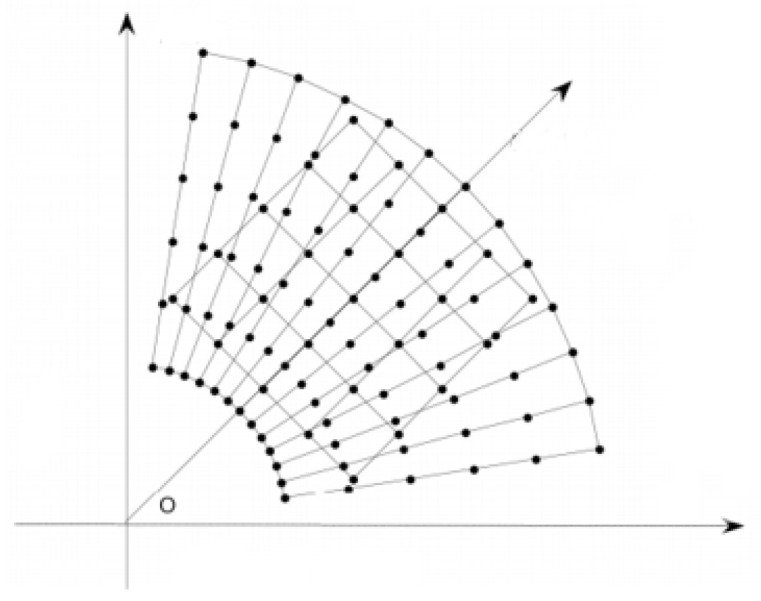
Interpolation diagram.

The spatial processing scale in the proposed method is shown in the area within the black line in Figure 5. Since the marine radar image is imaged in the polar coordinate system, the points close to the center of the image will be tightly arranged; conversely, the points far away from the center of the image will be sparsely arranged. Directly processing the fan-shaped region selected in the image will influence the effect of sea clutter suppression; thus, the data need to be converted to a rectangular coordinate system. This paper uses the nearest point interpolation method to interpolate the gray values of the points in the fan-shaped area into the grid in the rectangular coordinate system. The interpolation diagram is shown in Figure 6.

The polar coordinate of the point in the image is (r,θ,z), and the rectangular coordinate of the point is (*x*, *y*, *z*).

(18) $$\left\{\begin{array}{c} x=r^{*}\cos\theta \\ y=r^{*}\sin\theta \end{array}\right.$$

The coordinate of the point in the rectangular area is $\left(x_{0},y_{0}\right)$, and the polar coordinate $\left(r_{0},\theta_{0}\right)$ corresponding to the point is obtained by interpolation of the nearest point.

(19) $$\left\{\begin{array}{c} r_{0}=\mathrm{round}\left(\mathrm{sqrt}\left(x_{0}^{2}+y_{0}^{2}\right)\right)\\ \theta_{0}=\mathrm{round}\left(\mathrm{rem}\left(atan2\left(y_{0},x_{0}\right)+2\pi ,2\pi \right)\right) \end{array}\right.$$

### 4.2. Emd Sea Clutter Suppression Method for Marine Radar

EMD is an analysis method based on local characteristics in time domain proposed by Huang et al. in 1998 [60]. Since EMD has been used for marine radar applications in [63,71,72,73], and EMD is selected to be compared with the proposed method in this paper. It is an outstanding non-stationary and nonlinear signal analysis method. The residual function and intrinsic mode function (IMF) are generated by decomposing the time series signal. The latter has significant instantaneous frequency components, and the mean value of symmetrical local waveform is zero. The specific steps of the EMD algorithm are as follows:

(20) $$c_{1}\left(t\right)=\frac{H(t)+L(t)}{2}$$

where H(T) and L(T) are the upper envelope and lower envelope of the gray value time series S(T) of a single point. $c_{1}\left(t\right)$ is the average curve of the upper envelope and the lower envelope.

(21) $$r_{1}\left(t\right)=S\left(t\right)-c_{1}\left(t\right)$$

If $r_{1}\left(t\right)$ does not satisfies IMF conditions, define the average envelope of $r_{1}\left(t\right)$ as $c_{2}\left(t\right)$ and subtract $c_{2}\left(t\right)$ from $r_{1}\left(t\right)$.

(22) $$r_{2}\left(t\right)=r_{1}\left(t\right)-c_{2}\left(t\right)$$

This step is repeated many times until $r_{n}\left(t\right)$ satisfies the IMF condition. At this time, $r_{n}\left(t\right)$ is the residual function, which represents the trend of the signal, usually a constant or monotonic function.

(23) $$r_{n}\left(t\right)=S\left(t\right)-\sum_{j=1}^{n}c_{j}\left(t\right)$$

EMD sea clutter suppression method has been proved in [12] to have excellent sea clutter suppression effects when applied to longer time accumulation pulse radar. When it is applied to marine radar, the EMD method also has excellent suppression effects on sea clutter around the micro-motion target or floating target. In the meantime, the suppression performance of sea clutter around fast-moving targets will decrease, but it is still effective. At the same time, EMD will cyclically decompose the signal sequence of each range point; thus, it will spend more time on calculation.

### 4.3. Performance Evaluation Index

In order to evaluate the effect of the proposed method on sea clutter suppression and target detection, signal to noise ratio(SNR) [74] and detection probability PD are selected as performance evaluation indexes.

*SNR* is calculated as follows:

(24) $$\left(SNR\right)=10\log\left(\frac{s^{2}}{\langle x^{2}\rangle }\right)=10\log\left(\frac{{\langle s(n)\rangle }^{2}}{{\langle x(n)\rangle }^{2}}\right)$$

where *S* is the sum of signal gray values of all target points. *X* is the sum of signal gray value of all clutter points. 〈〉 represents the average calculation.

Another index, the detection rate PD of the target detector, is given in the form of the following [75]:

(25) $$P_{D}=\frac{N_{dt}}{N_{t}}$$

where $N_{t}$ is the total number of target points and $N_{dt}$ is the total number of detected target points.

### 4.4. Experimental Result

**Figure 7:**
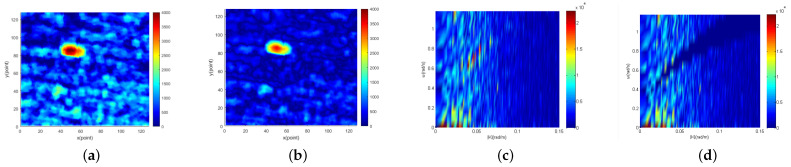
Low sea condition: (**a**) the 10th original image in radar image sequence; (**b**) the 10th sea clutter suppression image in radar image sequence; (**c**) 2D cross section of the 3D image spectrum of the original radar image sequence along |K|; (**d**) 2D cross section of the 3D image spectrum of the sea clutter suppressed radar image sequence along |K|.

**Figure 8:**
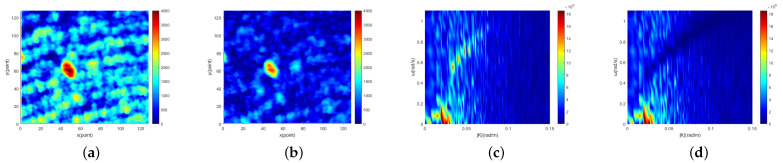
Medium sea condition: (**a**) the 3rd original image in radar image sequence; (**b**) the 3rd sea clutter suppression image in radar image sequence; (**c**) 2D cross section of the 3D image spectrum of the original radar image sequence along |K|; (**d**) 2D cross section of the 3D image spectrum of the sea clutter suppressed radar image sequence along |K|.

**Figure 9:**
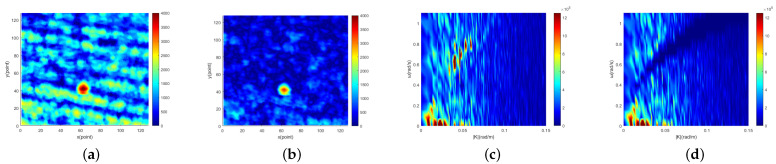
High sea condition: (**a**) The 10th original image in radar image sequence (**b**) The 10th sea clutter suppression image in radar image sequence (**c**) 2D cross section of the 3D image spectrum of the original radar image sequence along the |K| (**d**) 2D cross section of the 3D image spectrum of the sea clutter suppressed radar image sequence along the |K|.

Figure 7a, Figure 8a and Figure 9a show the single original image. Figure 7b, Figure 8b and Figure 9b show the corresponding single sea clutter suppressed image. The 2D cross-sections of the 3D image spectrum of the original image sequence along the |K| direction are shown in Figure 7c, Figure 8c and Figure 9c. In the meantime, the 2D cross-sections of the 3D image spectrum of the sea clutter suppressed image sequence along the |K| direction are shown in Figure 7d, Figure 8d and Figure 9d. In Figure 7, the sea condition level of the original image is low (1.03 m), and there are 207 target points in the image. The SNR of the original image is 16.8 db, and the SNR of the sea clutter suppressed image is 25.2 db. Thus, the SNR increased by 8.4 db. The sea condition level of the original image in Figure 8 is medium (1.87m), and there are 123 target points. The SNR of the original image is 16.6 db, and the SNR of the sea clutter suppressed image is 28.8 db. Hence, the SNR increased by 12.2 db. The sea condition level of the original image in Figure 9 is high (2.83m), and there are 75 target points. The original image SNR is 15.5 db, and the sea clutter suppressed image SNR is 30.3 db. Accordingly, SNR has increased by 14.8 db. In order to determine the target points and size of three targets, the following experiments were carried out. Firstly, Bayes, TCA and TOS CFAR detectors were used to detect them, and the basic shapes of the three targets were obtained. Then, by observing consecutive radar images, the first target (low sea condition) and the third target (high sea condition) were identified as isolated reefs, and the second target (medium sea condition) was a ship. Finally, combining the results of CFAR detection with the experience of manual observation, the target points of the three targets are determined. Accordingly, the range resolution of the experimental marine radar is 7.5 m, and the determined target points and the size of the three targets close to the real can be obtained. The length and width of the first target are approximately 150 m and 75 m, respectively. The length and width of the second target are about 135 m and 60 m, respectively. The third target is approximately 60 m and 75 m in length and width.

From the images obtained under three different sea conditions, it can be found that the sea clutter of the original image is overwhelmingly suppressed, and SNR is exceedingly improved. Furthermore, most of the suppressed sea clutter includes sea spike, because the energy gathered near the dispersion relationship is composed of sea spike energy. Removing sea peaks can not only improve image SNR but also reduce a lot of false alarms in subsequent target detection. With the increase in sea levels, the energy of the sea peak will also increase, and then suppressed energy will increase correspondingly.

**Figure 10:**
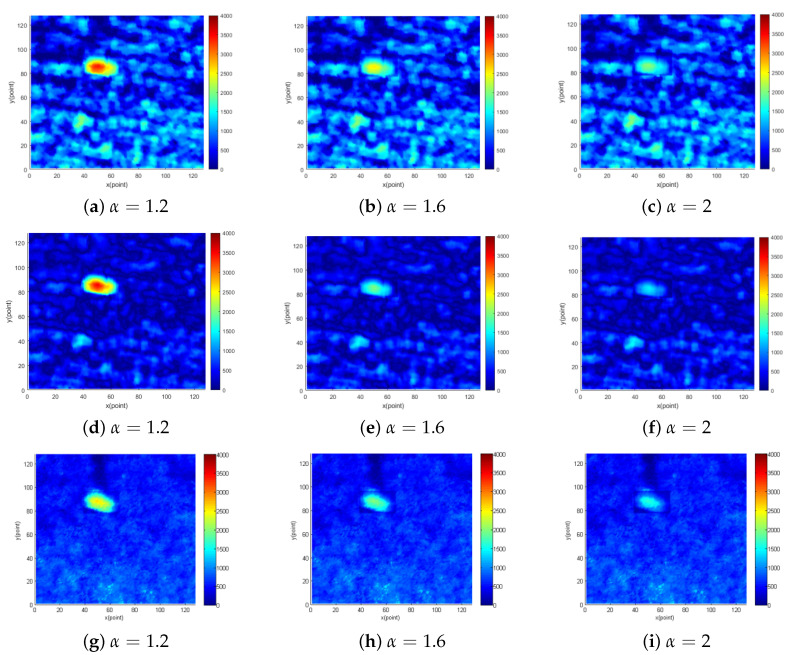
The 10th original image and corresponding suppression image in radar image sequence under low sea conditions.

In order to further verify the performance of the proposed method, three original image sequences selected above were artificially processed to reduce image SNR. Aiming at the target in the low sea condition, the gray value of the target points are reduced by α times. When the processing coefficient α is 1.2, 1.6 and 2.0, the original images obtained are shown in Figure 10a–c. The images of sea clutter suppressed by using the proposed method in this paper are shown in Figure 10d–f. The images of sea clutter suppressed by using the EMD method are shown in Figure 10g–i. It can be observed that the proposed method can effectively suppress most of the sea spikes in the image of different SNR. When SNR decreases to 13.5 db (α=1.2) and 8.0 db (α=1.6), the target points can be effectively retained, and SNR is separately increased by 7.5 db and 5.7 db. When SNR decreases to 3.7 db (α=2), since the points at the edge of the target are fully integrated into the sea clutter, some target points are suppressed as sea clutter. In this case, SNR increased by 3.8 db. The reason for the small SNR improvement is that the energy distribution of sea clutter is a slice of dispersed in low sea conditions. As can be seen, the inhibition effect of proposed method is basically consistent with the traditional EMD method, and in fact, the method still has a positive effect.

Table 2 shows the improvement effect of the proposed method and the EMD algorithm on the image SNR when α takes different values. As image SNR continues to decrease, the improvement of SNR will become smaller. The reason is that as the target gray value continues to decrease, the reduction value of the sea clutter gray value does not change. Then, the reason for no change is that the sea clutter in the original image has not changed; that is, the suppressed sea clutter energy is the same.

**Figure 11:**
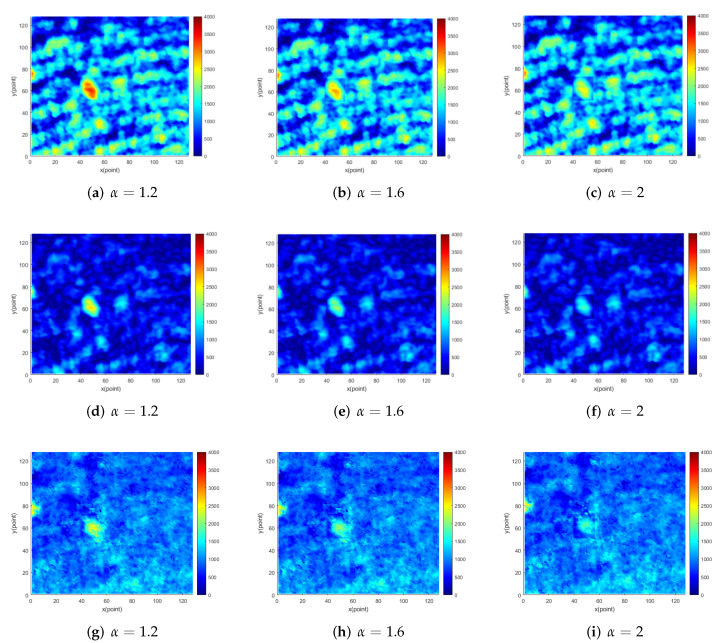
The 3rd original image and corresponding suppression image in radar image sequence under medium sea conditions.

**Figure 12:**
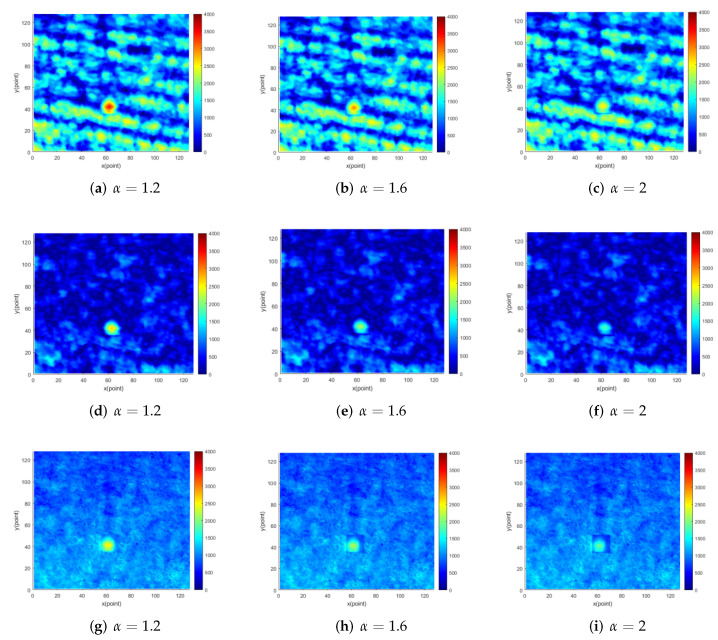
The 10th original image and corresponding suppression image in radar image sequence under high sea conditions.

Similarly, the target gray value is reduced by α times for the images under medium sea conditions and high sea conditions. Figure 11a–c and Figure 12a–c show the original images in the two cases. The sea clutter suppressed images by using the proposed method in two cases are shown in Figure 11d–f and Figure 12d–f. In addition, Figure 11g–i and Figure 12g–i show sea clutter suppressed images by using EMD method in two cases. In medium sea conditions and high sea conditions, the method can effectively suppress most of the sea spikes in the image and improve image SNR exceedingly. Table 3 and Table 4 show the improvement effect of the proposed method and the EMD method on the image SNR when α takes different values. In the same manner, the regular pattern of the method in this paper for sea clutter suppression under these two sea conditions is consistent with those of small sea conditions. In medium sea conditions, the method in this paper has a better suppression effect on sea clutter than that in low sea conditions. After the original SNR reaches 5 db, it can improve the SNR by at least 7.3 db. It has the best sea clutter suppression effect under high sea conditions and can improve the SNR of at least 8.5 db.

**Figure 13:**
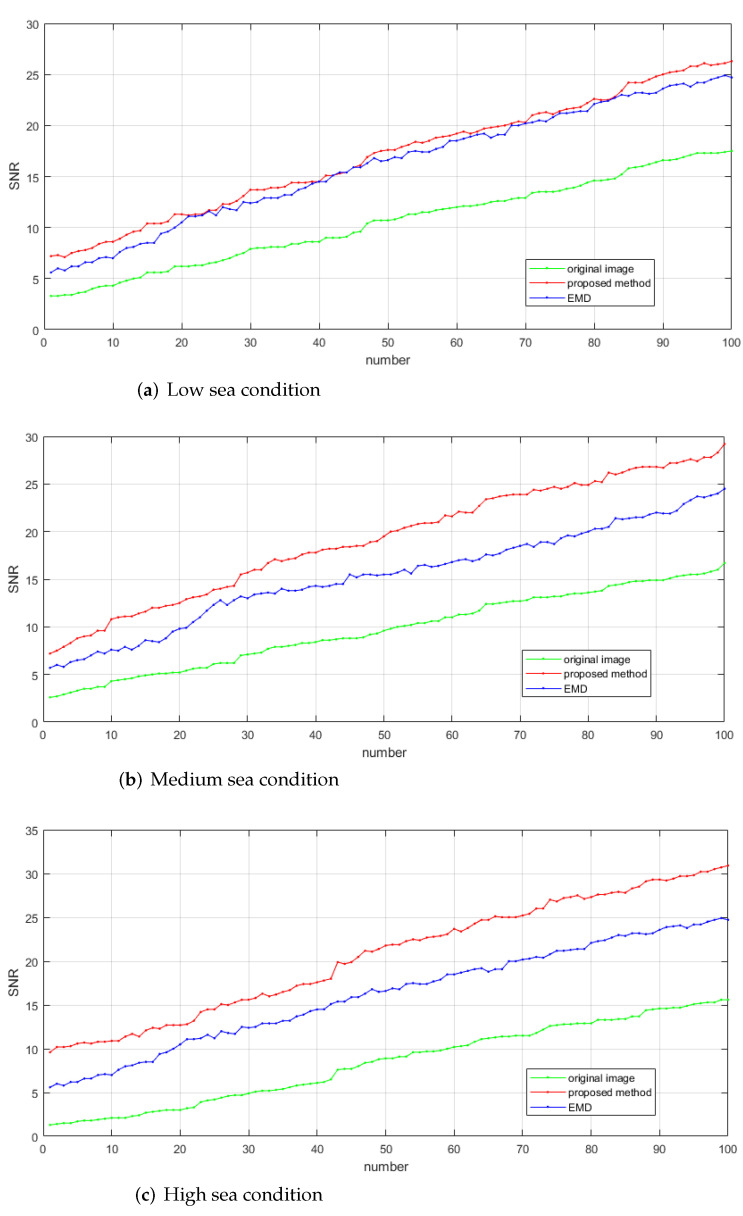
The suppression effect of EMD and the proposed method on sea clutter in three sea conditions of different SNR.

A total of 300 groups of real radar data of low sea condition, medium sea condition and high sea condition are selected for further verification. Figure 13 shows the results of the EMD method and the proposed method for improving SNR under low sea condition, medium sea condition and high sea condition. SNR has been sorted from small to large in the figure. Among them, the original image SNR in Figure 13a is between 3.3 db and 17.5 db, the original image SNR in Figure 13b is between 2.6 db and 16.7 db, and the original image SNR in Figure 13c is between 1.3 db and 15.6 db. Tthe performance advantages and disadvantages between proposed method and traditional EMD algorithm can be observed. In low sea conditions, although the performance of the two methods is basically the same, the proposed method not only can effectively improves the image SNR but also has a pleasurable suppression effect on sea clutter peaks. This provides positive conditions for subsequent target detection. Equally in the medium sea condition, compared to the EMD method, the proposed method has obvious advantages in improving the image SNR. Finally in high sea conditions, the performance of the proposed method is much better than that of the traditional EMD method, and it can perfectly improve the image SNR.

As described, a large number of experimental results show that the proposed method can effectively suppress sea clutter and perfectly improve SNR under different SNR of different sea condition levels. The increased maximum SNR is 8.8 db, 12.5 db and 15.3 db in low sea condition, medium sea condition and high sea condition. As observed, the effect is remarkable.

Next, WL-CFAR is used to detect the target in the original image and the sea clutter suppressed image. The proposer of WL-CFAR provides the selection basis of each parameter of WL-CFAR in [7]. The value of the shape parameter *C* of the Weibull distribution is set according to the distribution characteristics of the clutter data, usually between 1 and 2. In order to determine the value of *C*, firstly, the probability density function curve of the experimental data is fitted. Then, the fitted curve is compared with the Weibull distribution curves for which its shape parameter is 1, 1.1,..., 2. The performance evaluation method is KL distance measurement and KS test. Finally, select the shape parameter when KL and KS are the smallest as the value of *C*. In order to achieve around 16 false alarm points in each image and stabilize the detection rate at a high level, PFA is set to 0.001. Since the threshold τ is determined according to PFA [7], τ is set to 3. Based on the three sliding window scales of 16, 24 and 32 commonly used in marine radar target detection [5,6], the sliding window scale *N* of WL-CFAR is set to these three sizes, and then a large number of comparisons are made by using experimental data. The results show that when the sliding window scale *N* is 32, the WL-CFAR detector has the best detection effect on the experimental data. The value of robustness measurement parameter β is proved to be the best set between 0.1 and 0.9. Moreover the smaller the β value, the better the detector’s robustness [7]. After other parameters are determined, the value of β is set to 0.1, 0.2,..., 0.9, respectively, and WL-CFAR with the set β value is used to detect the target of our experimental data. As described above, WL-CFAR has the best detection when the β value is 0.1. Therefore, in this paper, PFA is set to 0.001, and τ is set to 3. Then, *C* is set to 1, and the sliding window scale *N* is set to 32. Then, β is set to 0.1. Under different sea conditions and different SNR, the detection probabilities of the sea clutter suppressed image and the original image are given in Table 5, Table 6 and Table 7.

It can be observed that the detection probability increased by an average of 8% in low sea conditions. Meanwhile, the probability of detection increased by 26% on average in medium sea conditions. Moreover, in high sea conditions, the detection probability increased by 37% on average. This is due to the excellent sea clutter suppression method, which can effectively remove sea peaks causing false alarms. The higher the sea condition level is, the more obvious the effect of sea clutter suppression, and ultimately the greater the probability of improved target detection. It can be concluded that the propose method has a higher detection rate for weak targets in complex sea conditions.

## 5. Conclusions

The wave inversion technology was applied to the field of target detection, and a new target detection algorithm under sea clutter suppression for marine radar was proposed. The effectiveness of the method was verified by 300 groups of real radar data under three different sea conditions. The main innovations and conclusions are as follows:

1. A sea clutter suppression link is added before the detecting target. The leading wavelength dispersion relation is introduced into the target detection of marine radar for the first time, and a sea clutter suppression and target detection algorithm of marine radar image sequence based on spatio-temporal joint filtering was proposed;
2. Compared with the traditional EMD sea clutter suppression method, the spatio-temporal combined sea clutter suppressor proposed in this paper can effectively suppress the sea spikes in the image and can increase SNR by 15.3 db at most, which is more than 8.6 db of the EMD method;
3. Compared with the WL-CFAR without sea clutter suppression method, the detection algorithm under sea clutter suppression proposed in this paper can excellently improve the detection probability of weak targets in complex sea conditions, and the probability can be up to 37% at most.

## Figures and Tables

**Table 1:** X-band marine radar parameters.

Radar Parameters	The Performance
Electromagnetic Wave Frequency	9.4 Ghz
Antenna Angular Speed	26 r.p.m
Antenna Height	25 m
Polarization	HH
Range Resolution	7.5 m
Horizontal Beam Width	1.3°
Vertical Beam Width	23°
Pulse Repetiton Frequency	1300 Hz
Pulse Width	0.7°

**Table 2:** Comparison of SNR of two methods under low sea conditions.

Proposed Algorithm			EMD		
Processing Coefficient α	Original Image SNR (*db*)	Suppressed Image SNR (*db*)	SNR Improvement (*db*)	Suppressed Image SNR (*db*)	SNR Improvement (*db*)
1	16.8	25.2	8.4	23.3	8.0
1.2	13.5	21.0	7.5	18.4	7.2
1.4	10.6	17.0	6.4	15.1	6.1
1.6	8.0	13.7	5.7	12.7	5.3
1.8	5.7	10.5	4.8	9.9	4.4
2	3.6	7.4	3.8	7.9	3.3

**Table 3:** Comparison of SNR of two methods under medium sea conditions.

Proposed Algorithm			EMD		
Processing Coefficient α	Original Image SNR (*db*)	Suppressed Image SNR (*db*)	SNR Improvement (*db*)	Suppressed Image SNR (*db*)	SNR Improvement (*db*)
1	16.6	28.8	12.2	24.9	7.3
1.2	13.0	24.4	11.3	20.2	6.2
1.4	10.0	20.0	10	16.3	5.3
1.6	7.4	15.7	8.3	12.9	4.5
1.8	5.0	12.3	7.3	9.7	3.7
2	2.9	7.5	4.6	6.8	2.9

**Table 4:** Comparison of SNR of two methods under high sea conditions.

Proposed Algorithm			EMD		
Processing Coefficient α	Original Image SNR (*db*)	Suppressed Image SNR (*db*)	SNR Improvement (*db*)	Suppressed Image SNR (*db*)	SNR Improvement (*db*)
1	15.5	30.3	14.8	24.1	8.6
1.2	11.9	25.6	13.7	19.8	7.9
1.4	8.8	21.3	12.5	15.8	7.0
1.6	6.2	17.4	11.2	12.4	6.2
1.8	3.8	13.7	9.9	9.2	5.4
2	1.7	10.2	8.5	6.1	4.4

**Table 5:** Target detection results in low sea conditions.

PD(%)						
original image SNR	3.6 (db)	5.7 (db)	8.0 (db)	10.6 (db)	13.5 (db)	16.8 (db)
proposed method	37.2	58.3	71.1	83.3	90.0	97.8
original image	29.3	52.2	63.9	75.0	81.1	88.3

**Table 6:** Target detection results in medium sea conditions.

PD(%)						
original image SNR	2.9 (db)	5.0 (db)	7.4 (db)	10.0 (db)	13.0 (db)	16.6 (db)
proposed method	23.4	40.9	62.8	77.6	86.1	97.1
original image	0.0	10.2	21.0	40.2	77.3	80.6

**Table 7:** Target detection results in high sea conditions.

PD(%)						
original image SNR	1.7 (db)	3.8 (db)	6.2 (db)	8.8 (db)	11.9 (db)	15.5 (db)
proposed method	25.3	36.0	52.0	64.0	90.7	97.3
original image	0.0	0.0	0.0	18.7	48.0	77.3

## Data Availability

The raw/processed data required to reproduce these findings cannot be shared at this time as the data also forms part of an ongoing study.

## References

[1] Chen S., Huang W. (2013). Maneuvering Target Tracking from Nautical Radar Images Using Particle-Kalman Filters. J. Electromagn. Waves Appl..

[2] Mark A.R., James A.S., William A.H. (2010). Principles of Modern Radar: Basic Principles.

[3] Watts S. (1996). Cell-Averaging Cfar Gain in Spatially Correlated K-Distributed Clutter. IEE Proc.: Radar Sonar Navig..

[4] Rohling H. (1983). Radar Cfar Thresholding in Clutter and Multiple Target Situations. IEEE Trans. Aerosp. Electron. Syst..

[5] Gandhi P.P., Saleem A.K. (1988). Analysis of Cfar Processors in Nonhomogeneous Background. IEEE Trans. Aerosp. Electron. Syst..

[6] Weinberg G.V. (2020). Interference Control in Sliding Window Detection Processes Using a Bayesian Approach. Digit. Signal Process..

[7] Zhang B., Zhou J., Xie J., Zhou W. (2021). Weighted Likelihood Cfar Detection for Weibull Background. Digit. Signal Process..

[8] Chen S., Huang W. Target Tracking Using Particle Filter with X-Band Nautical Radar. Proceedings of the 2013 IEEE Radar Conference (RadarCon13).

[9] Chen S. (2014). Particle Filter Based Target Tracking from X-band Nautical Radar Images. Master’s Thesis.

[10] Yang Y., Xiao S.-P., Wang X.-S. (2018). Radar Detection of Small Target in Sea Clutter Using Orthogonal Projection. IEEE Geosci. Remote Sens. Lett..

[11] Guo L., Deng W., Yao D., Yang Q., Ye L., Zhang X. (2021). A Knowledge-Based Auxiliary Channel Stap for Target Detection in Shipborne Hfswr. Remote Sens..

[12] Lv M., Zhou C. (2019). Study on Sea Clutter Suppression Methods Based on a Realistic Radar Dataset. Remote Sens..

[13] Shang S., He K.-N., Wang Z.-B., Yang T., Liu M., Li X. (2020). Sea Clutter Suppression Method of Hfswr Based on Rbf Neural Network Model Optimized by Improved Gwo Algorithm. Comput. Intell. Neurosci..

[14] Li G.-q., Song Z.-Y., Fu Q. (2020). A Convolutional Neural Network Based Approach to Sea Clutter Suppression for Small Boat Detection. Front. Inf. Technol. Electron. Eng..

[15] Lee M.-J., Lee S.-J., Ryu B.-H., Lim B.-G., Kim K.-T. (2020). Reduction of False Alarm Rate in Sar-Mti Based on Weighted Kurtosis. IEEE Trans. Geosci. Remote Sens..

[16] Xiang H., Zhang Y., Zhang L., Chen Z., Zhang L. (2022). Detection and Fast Motion Parameter Estimation for Target with Range Walk Effect Based on New Axis Rotation Moving Target Detection. Digit. Signal Process..

[17] Yang J., Qiu X., Shang M., Zhong L., Ding C. (2020). A Method of Marine Moving Targets Detection in Multi-Channel Scansar System. Remote Sens..

[18] He Z., Li Z., Chen X., Yu A., Yi T., Dong Z. (2021). Detecting Moving Target on Ground Based on Its Shadow by Using Videosar. Remote Sens..

[19] Jaya E., Krishna B.T. (2020). A Particle Fuzzy Decisive Framework for Moving Target Detection in the Multichannel Sar Framework. Int. J. Comput. Intell. Appl..

[20] Jaya E., Krishna B.T. (2020). Fuzzy-Based Mtd: A Fuzzy Decisive Approach for Moving Target Detection in Multichannel Sar Framework. Data Technol. Appl..

[21] Wang H., Tang Z., Zhao Y., Chen Y., Zhu Z., Zhang Y. (2019). Signal Processing and Target Fusion Detection Via Dual Platform Radar Cooperative Illumination. Sensors.

[22] Geng Z., Motahari S.M.A., Deng H., Himed B. (2018). Ground Moving Target Detection for Airborne Radar Using Clutter Doppler Compensation and Digital Beamforming. Microw. Opt. Technol. Lett..

[23] Huang P., Zou Z., Xia X.-G., Liu X., Liao G., Xin Z. (2021). Multichannel Sea Clutter Modeling for Spaceborne Early Warning Radar and Clutter Suppression Performance Analysis. IEEE Trans. Geosci. Remote Sens..

[24] Zhang T., Gu H., Su W., Tan K. (2021). Ground Moving Target Indication and Parameter Estimation Algorithm Using Deramp Space-Time Adaptive Processing. J. Appl. Remote Sens..

[25] Ślesicka A., Kawalec A. (2020). An Application of the Orthogonal Matching Pursuit Algorithm in Space-Time Adaptive Processing. Sensors.

[26] Blasone G.P., Colone F., Lombardo P., Wojaczek P., Cristallini D. (2021). Dual Cancelled Channel STAP for Target Detection and DOA Estimation in Passive Radar. Sensors.

[27] Root B.T. (1998). Hf-over-the-Horizon Radar Ship Detection with Short Dwells Using Clutter Cancelation. Radio Sci..

[28] Root B.T. Hf Radar Ship Detection through Clutter Cancellation. Proceedings of the 1998 IEEE Radar Conference Challenges in Radar Systems and Solutions (Cat. No. 98CH36197), RADARCON’98.

[29] Quan Y., Zhang L., Li Y., Wang H., Xing M. (2014). Othr Spectrum Reconstruction of Maneuvering Target with Compressive Sensing. Int. J. Antennas Propag..

[30] Pan M., Chen J., Xie S., Wang S. (2019). Sea Clutter Suppression Algorithm Based on Signal Resonance. J. Eng..

[31] Zhao W., Chen Z., Jin M. (2020). Subband Maximum Eigenvalue Detection for Radar Moving Target in Sea Clutter. IEEE Geosci. Remote Sens. Lett..

[32] Wang Y., Wu D., Yu Q., Zhu D., Meng F. (2021). A Weather Signal Detection Algorithm Based on Evd in Elevation for Airborne Weather Radar. Digit. Signal Process..

[33] Al-Jazzar S.O., Aldalahmeh S. (2018). Aoa, Delay, and Complex Propagation Factor Estimation for the Monostatic Mimo Radar System. Int. J. Antennas Propag..

[34] Redif S., Weiss S., McWhirter J.G. (2017). Relevance of Polynomial Matrix Decompositions to Broa dband Blind Signal Separation. Signal Process..

[35] Choi S., Yang H., Song J., Jeon H., Kim J., Chung Y. (2021). Sea Clutter Covariance Matrix Estimation and Its Application to Whitening Filter. J. Electromagn. Eng. Sci..

[36] Ren H., Feng X. (2020). Calculating Vertical Deformation Using a Single Insar Pair Based on Singular Value Decomposition in Mining Areas. Int. J. Appl. Earth Obs. Geoinf..

[37] Xiong Y., Liang B., Yu H., Chen J., Jin Y., Xing M. (2021). Processing of Bistatic Sar Data with Nonlinear Trajectory Using a Controlled-Svd Algorithm. IEEE J. Sel. Top. Appl. Earth Obs. Remote Sens..

[38] Xu X., Feng C., He S. (2020). A Method for the Micro-Motion Signal Separation and Micro-Doppler Extraction for the Space Precession Target. IEEE Access.

[39] Yan H., Chen C., Jin G., Zhang J., Wang X., Zhu D. (2021). Implementation of a Modified Faster R-Cnn for Target Detection Technology of Coastal Defense Radar. Remote Sens..

[40] Baird Z., Mcdonald M.K., Rajan S., Lee S.J. (2020). A Cnn-Lstm Network for Augmenting Target Detection in Real Maritime Wide Area Surveillance Radar Data. IEEE Access.

[41] Gonzales-Martínez R., Machacuay J., Rotta P., Chinguel C. (2022). Hyperparameters Tuning of Faster R-Cnn Deep Learning Transfer for Persistent Object Detection in Radar Images. IEEE Lat. Am. Trans..

[42] Vondra B., Bonefacic D. (2020). Mitigation of the Effects of Unknown Sea Clutter Statistics by Using Radial Basis Function Network. Radioengineering.

[43] Pandeeswari B., Sutha J., Parvathy M. (2021). A Novel Synthetic Aperture Radar Image Change Detection System Using Radial Basis Function-Based Deep Convolutional Neural Network. J. Ambient Intell. Hum. Comput..

[44] Shen Y., Liu M., Wang J., Zhou W. A New Image Restoration Algorithm Based on Mathematical Morphology and Wavelet Neural Network. Proceedings of the 2012 International Conference on Computer Application and System Modeling.

[45] Cao C., Hou Q., Gulliver T.A., Lan Q. (2020). A Passive Detection Algorithm for Low-Altitude Small Target Based on a Wavelet Neural Network. Soft Comput..

[46] FallahReyhani M., Bakhshi H., Lohrasbipeyde H. (2021). Doa Estimation of Lfm Signal Based on Krylov Subspace Method. Telecommun. Syst..

[47] Park D., Lee S., Park S., Kwak N. (2021). Radar-Spectrogram-Based Uav Classification Using Convolutional Neural Networks. Sensors.

[48] Wang Q., Chen Z., Zhou Q., Wu X. (2020). Mitigation of Radio Frequency Interference in Hfswr Using Fractional Fourier Transform Based Filtering Algorithms. IEEE Geosci. Remote Sens. Lett..

[49] Gao C., Tao R., Kang X. (2020). Weak Target Detection in the Presence of Sea Clutter Using Radon-Fractional Fourier Transform Canceller. IEEE J. Sel. Top. Appl. Earth Obs. Remote Sens..

[50] Gaglione D., Clemente C., Ilioudis C.V., Persico A.R., Proudler I.K., Soraghan J.J., Farina A. (2018). Waveform Design for Communicating Radar Systems Using Fractional Fourier Transform. Digit. Signal Process..

[51] Campbell J.B., Pérez F., Wang Q., Santhanam B., Dunkel R., Doerry A.W., Atwood T., Hayat M.M. (2018). Remote Vibration Estimation Using Displaced-Phase-Center Antenna Sar for Strong Clutter Environments. IEEE Trans. Geosci. Remote Sens..

[52] Filip-Dhaubhadel A., Shutin D. (2020). Long Coherent Integration in Passive Radar Systems Using Super-Resolution Sparse Bayesian Learning. IEEE Trans. Aerosp. Electron. Syst..

[53] Zhang H., Shan T., Liu S., Tao R. (2021). Performance Evaluation and Parameter Optimization of Sparse Fourier Transform. Signal Process..

[54] Chen X., Yu X., Huang Y., Guan J. (2020). Adaptive Clutter Suppression and Detection Algorithm for Radar Maneuvering Target with High-Order Motions Via Sparse Fractional Ambiguity Function. IEEE J. Sel. Top. Appl. Earth Obs. Remote Sens..

[55] Yu X., Chen X., Huang Y., Guan J. (2019). Fast Detection Method for Low-Observable Maneuvering Target Via Robust Sparse Fractional Fourier Transform. IEEE Geosci. Remote Sens. Lett..

[56] Cai J., Zhou H., Huang W., Wen B. (2020). Ship Detection and Direction Finding Based on Time-Frequency Analysis for Compact Hf Radar. IEEE Geosci. Remote Sens. Lett..

[57] Yang Z., Tang J., Zhou H., Xu X., Tian Y., Wen B. (2021). Joint Ship Detection Based on Time-Frequency Domain and Cfar Methods with Hf Radar. Remote Sens..

[58] Yang Z., Zhou H., Tian Y., Huang W., Shen W. (2021). Improving Ship Detection in Clutter-Edge and Multi-Target Scenarios for High-Frequency Radar. Remote Sens..

[59] Yang Z., Zhou H., Tian Y., Zhao J. (2021). Improved Cfar Detection and Direction Finding on Time-Frequency Plane with High-Frequency Radar. IEEE Geosci. Remote Sens. Lett..

[60] Huang N.E., Shen Z., Long S.R. (1999). A New View of Nonlinear Water Waves: The Hilbert Spectrum. Annu. Rev. Fluid Mech..

[61] Xiao C., Hao C., Dong X. Sea Clutter Characteristics Analysis and Target Detection Based on Hht. Proceedings of the 2011 International Conference on Consumer Electronics, Communications and Networks (CECNet).

[62] Li Z., Zhu Y., Fu Q. Weak Target Detection Based on EMD and Hurst Exponent. Proceedings of the 8th International Conference on Digital Image Processing (ICDIP).

[63] Wang Y., Wei Y., Chao L.G., Kang W. Analysis of Spectrum Characteristics of the Acquired Sea Clutter Data Based on HHT. Proceedings of the 2014 IEEE Symposium on Computer Applications and Communications.

[64] Briggs J.N. (2005). Target Detection by Marine Radar. IEEE Aerosp. Electron. Syst. Mag..

[65] Young I.R., Rosenthal W., Ziemer F. (1985). A Three-Dimensional Analysis of Marine Radar Images for the Determination of Ocean Wave Directionality and Surface Currents. J. Geophys. Res. Oceans.

[66] Wei Y., Zheng Y., Lu Z. (2020). A Method for Retrieving Wave Parameters from Synthetic X-Band Marine Radar Images. IEEE Access.

[67] Wei Y., Zhang J.-K., Lu Z. (2016). A Novel Successive Cancellation Method to Retrieve Sea Wave Components from Spatio-Temporal Remote Sensing Image Sequences. Remote Sens..

[68] Nieto-Borge J.C., Hessner K., Jarabo-Amores P., Mata-Moya D.D.L. (2008). Signal-to-Noise Ratio Analysis Heights from X-Band Marine to Estimate Ocean Wave Radar Image Time Series. IET Radar Sonar Navig..

[69] Reichert K., Hessner K., Dannenberg J., Tränkmann I. X-Band Radar as a Tool to Determine Spectral and Single Wave Properties. Proceedings of the International Conference on Offshore Mechanics and Arctic Engineering 2006.

[70] Stevens C.L., Poulter E.M., Smith M.J., McGregor J.A. (1999). Nonlinear Features in Wave-Resolving Microwave Radar Observations of Ocean Waves. IEEE. Oceanic Eng..

[71] Huang W., Liu X., Gill E.W. (2017). An Empirical Mode Decomposition Method for Sea Surface Wind Measurements from X-Band Nautical Radar Data. IEEE Trans. Geosci. Remote Sens..

[72] Liu X., Huang W., Gill E.W. (2017). Wind Direction Estimation from Rain-Contaminated Marine Radar Data Using the Ensemble Empirical Mode Decomposition Method. IEEE Trans. Geosci. Remote Sens..

[73] Liu X., Huang W., Gill E.W. (2017). Estimation of Significant Wave Height from X-Band Marine Radar Images Based on Ensemble Empirical Mode Decomposition. IEEE Geosci. Remote Sens. Lett..

[74] Sayama S. (2021). Detection of Target and Suppression of Sea and Weather Clutter in Stormy Weather by Weibull/CFAR. IEEJ Trans. Electr. Electron. Eng..

[75] Lee M.-J., Kim J.-E., Ryu B.-H., Kim K.-T. (2021). Robust Maritime Target Detector in Short Dwell Time. Remote Sens..

